# Super-resolution synthetic MRI using deep learning reconstruction for accurate diagnosis of knee osteoarthritis

**DOI:** 10.1186/s13244-025-01911-z

**Published:** 2025-02-17

**Authors:** Kejun Wang, Weiyin Vivian Liu, Renjie Yang, Liang Li, Xuefang Lu, Haoran Lei, Jiawei Jiang, Yunfei Zha

**Affiliations:** 1 Department of Radiology, Renmin Hospital of Wuhan University, Wuhan, China; 2 MR Research, GE Healthcare, Beijing, China; 3 School of Computer Science, Wuhan University, Wuhan, China

**Keywords:** Knee, Osteoarthritis, Magnetic resonance imaging, Cartilage, T2

## Abstract

**Objective:**

To assess the accuracy of deep learning reconstruction (DLR) technique on synthetic MRI (SyMRI) including T2 measurements and diagnostic performance of DLR synthetic MRI (SyMRI_DL_) in patients with knee osteoarthritis (KOA) using conventional MRI as standard reference.

**Materials and methods:**

This prospective study recruited 36 volunteers and 70 patients with suspected KOA from May to October 2023. DLR and non-DLR synthetic T2 measurements (T2-SyMRI_DL_, T2-SyMRI) for phantom and in vivo knee cartilage were compared with multi-echo fast-spin-echo (MESE) sequence acquired standard T2 values (T2_MESE_). The inter-reader agreement on qualitative evaluation of SyMRI_DL_ and the positive percent agreement (PPA) and negative percentage agreement (NPA) were analyzed using routine images as standard diagnosis.

**Results:**

DLR significantly narrowed the quantitative differences between T2-SyMRI_DL_ and T2_MESE_ for 0.8 ms with 95% LOA [−5.5, 7.1]. The subjective assessment between DLR synthetic MR images and conventional MRI was comparable (all *p* > 0.05); Inter-reader agreement for SyMRI_DL_ and conventional MRI was substantial to almost perfect with values between 0.62 and 0.88. SyMRI_DL_ MOAKS had substantial inter-reader agreement and high PPA/NPA values (95%/99%) using conventional MRI as standard reference. Moreover, T2-SyMRI_DL_ measurements instead of non-DLR ones significantly differentiated normal-appearing from injury-visible cartilages.

**Conclusion:**

DLR synthetic knee MRI provided both weighted images for clinical diagnosis and accurate T2 measurements for more confidently identifying early cartilage degeneration from normal-appearing cartilages.

**Critical relevance statement:**

One-acquisition synthetic MRI based on deep learning reconstruction provided an accurate quantitative T2 map and morphologic images in relatively short scan time for more confidently identifying early cartilage degeneration from normal-appearing cartilages compared to the conventional morphologic knee sequences.

**Key Points:**

- Deep learning reconstruction (DLR) synthetic knee cartilage T2 values showed no difference from conventional ones.
- DLR synthetic T1-, proton density-, STIR-weighted images had high positive percent agreement and negative percentage agreement using MRI OA Knee Score features.
- DLR synthetic T2 measurements could identify early cartilage degeneration from normal-appearing ones.

**Graphical Abstract:**

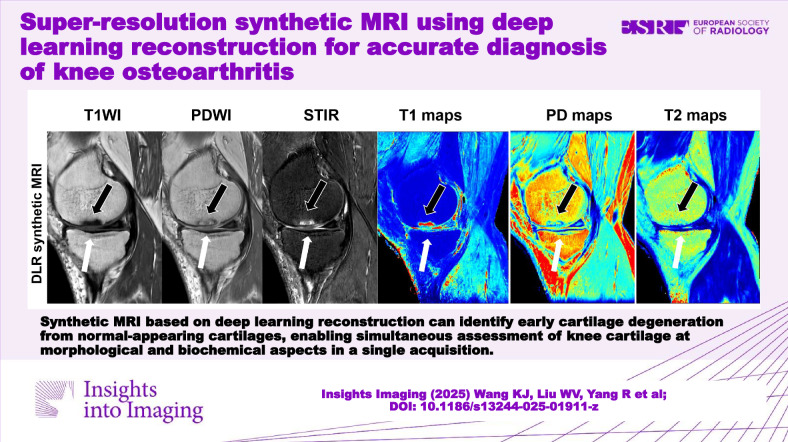

## Introduction

The early detection of knee osteoarthritis (KOA) needs an effective and efficient imaging method. Early non-surgical interventions in cartilage degeneration in normal-appearing cartilage, such as weight loss, aerobic activity, and musculoskeletal exercises of extension and flexion may alleviate symptoms of joint degeneration, slow progression, and reduce the number of OA patients who are required to undergo joint replacement surgery [1]. Non-invasive MRI-based quantitative imaging can detect changes in cartilage compositions and fine structures, even before the onset of morphological cartilage loss [2]. T2 mapping involved in a routine MRI knee protocol has been commonly applied for the detection of cartilage lesions, especially significantly improved identification of early cartilage degeneration [1]. It is sensitive to the water content, concentration of Type II collagen, and anisotropic tissue structures in cartilages [3, 4].

Several factors, including scan time, poor spatial resolution, and robustness and accuracy of measurements, have hindered applications of quantitative MRI in clinical practice [5]. A promising approach to address these limitations is synthetic magnetic resonance imaging (synthetic MRI), using two echo times (TE) and four delay times in a spin-echo sequence with a 120° saturation radiofrequency (RF) pulse followed by 90–180° excitation RF pulses. This single acquisition generates quantitative MRI (qMRI) including T1, proton density (PD), and T2 maps as well as countless weighted images. By adjusting inversion time (TI), repetition time (TR), and TE, it can either “enhance” or “suppress” signals of a specific tissue, such as fat, in the synthetic weighted images [6]. Additionally, double-inversion recovery (DIR) with synthetic MRI allows to diagnose knee synovitis in patients who are allergic to gadolinium contrast agents [7]. Synthetic MRI provides different tissue contrast images for diagnosing joint diseases, enabling one-stop assessment of structural lesions and early cartilage damage in the knee [8]. To find a trade-off between acquisition time and image quality, larger slice thickness and gap are usually set for synthetic T2 knee images compared to conventional ones, potential leading to partial volume effects that may affect accuracy and confidence of knee diagnosis. On the other hand, to set the same in-plane spatial resolution, slice thickness and gap for synthetic T2 knee images as conventional T2-weighted imaging sequence increases noise, worsens image quality, and doubles the scan time. Therefore, synthetic MRI still faces challenges, such as inadequate image quality, insufficient spatial resolution, the presence of artifacts and a lower detection rate for cartilage lesions and ligament injury [6, 9].

A deep learning reconstruction (DLR) technique breaks the tradeoffs between signal-to-noise ratio, spatial resolution, and scan time [10]. The commercial inline optimized CNN algorithm is a data-driven end-to-end approach that preserves fidelity and removes noises and Gibbs artifacts before Fourier transformation to reconstruct images [11, 12]. Except image quality and diagnostic performance of DLR conventional MR imaging [12], the effect of DLR on knee synthetic MRI regarding qualitative and quantitative evaluation has not yet been studied. Our study aimed to: (1) evaluate the impact of DLR technique on the synthetic T2 measurement for phantom and volunteers, using multi-echo spin-echo (MESE) sequence as the standard reference; (2) explore the diagnostic performance of DLR synthetic MRI in patients with KOA compared to conventional MRI.

## Materials and methods

### Phantom study

2D synthetic MR images were acquired using a multi-dynamic, multi-echo sequence (MAGnetic resonance Imaging Compliant, MAGiC) on 3.0-T MRI (Signa Architect; GE Healthcare) equipped with an 18-channel knee coil. The synthetic images were reconstructed using conventional reconstruction (SyMRI) and a commercial inline deep-learning reconstruction (DLR, brand name: AIR^TM^ Recon DL, MR30.1_R01, GE Healthcare, USA), hereafter referred to as SyMRI_DL_. AIR^TM^ Recon DL leverages rectified linear unit activations without bias terms in a convolution neural network (CNN) to analyze the raw image data directly, aiming to minimize noise and artifacts [10, 12, 13]. A “high” denoising level was applied to a Hann window threshold to reconstruct image.

To validate T2 estimation, a home-made phantom was imaged, comprising five tubes with aqueous CuSO_4_ (anhydrous copper sulfate; purity 97.5%; Tianjin JVHENGDA Co., China) at concentrations of 13, 15, 20, 25 and 27 mM that simulate T2 values within a range of approximately 25 to 70 ms. On both SyMRI and SyMRI_DL_ computed T2 quantitative maps, T2 values (T2-SyMRI and T2-SyMRI_DL_) were measured using a region of interest (ROI) with a 25-mm diameter within each tube with a 28-mm diameter by a single reader (R.Y.). A multi-echo spin-echo (MESE) sequence with six echo times (14.4, 28.8, 43.2, 57.7, 72.1, and 86.5 ms) served as the reference standard for T2 measurements (T2_MESE_). The detailed acquisition parameters for both MAGiC and MESE sequences are shown in Table 1.

**Table 1:** Parameters of the synthetic MRI sequence, the conventional morphologic and multi-echo spin-echo T2 mapping sequence

Technical parameter	Synthetic MRI	MESE	Conventional MRI				
			T1-weighted	PD-weighted	STIR	FS IW	FS IW
Plane	Sagittal	Sagittal	Sagittal	Sagittal	Sagittal	Coronal	Transverse
No. of section	28	28	28	28	28	24	28
Thickness/gap (mm)	3/0.3	3/0.3	3/0.3	3/0.3	3/0.3	3/0.3	3/0.3
Field of view	160 × 160	160 × 160	160 × 160	160 × 160	160 × 160	160 × 160	160 × 160
Acquisition matrix	320 × 256	320 × 256	320 × 256	320 × 256	320 × 256	384 × 256	320 × 256
Voxel size (mm)	0.5 × 0.6 × 3	0.5 × 0.6 × 3	0.5 × 0.6 × 3	0.5 × 0.6 × 3	0.5 × 0.6 × 3	0.4 × 0.6 × 3	0.5 × 0.6 × 3
Fat saturation	Off	Off	Off	Off	Off	On	On
AIR^TM^ Recon DL strength	Off/high	/	Off	Off	Off	Off	Off
TR (ms)	4000	1200	480	1500	4800	2938	2720
TE (ms)	17.2, 86.0	14.4, 28.8, 43.2, 57.7, 72.1, 86.5	6.1	28	42	41	36
Acceleration	2	2	2	2	2	2	2
Acq. time (min:s)	4:32	6:18	1:45	2:49	3:02	1:06	1:13

*STIR* short-tau inversion recovery, *TI* 220 ms, *FS IW* fat-saturated intermediate weighted, *MESE* multi-echo spin echo

### Subjects

This study was approved by our institutional ethics committee and prospectively recruited 36 healthy volunteers and 70 patients with suspected knee OA from May 2023 to October 2023. All participants without MRI contraindications signed an informed consent form before enrolling in our study. In a random order, all healthy subjects underwent synthetic MRI as well as MESE sequence while all patients with suspected KOA underwent a conventional protocol comprising five sequences (sagittal T1WI/PDWI, sagittal, axial, and coronal fat-suppressed IW sequences) and a sagittal SyMRI sequence (Table 1).

**Fig. 1:**
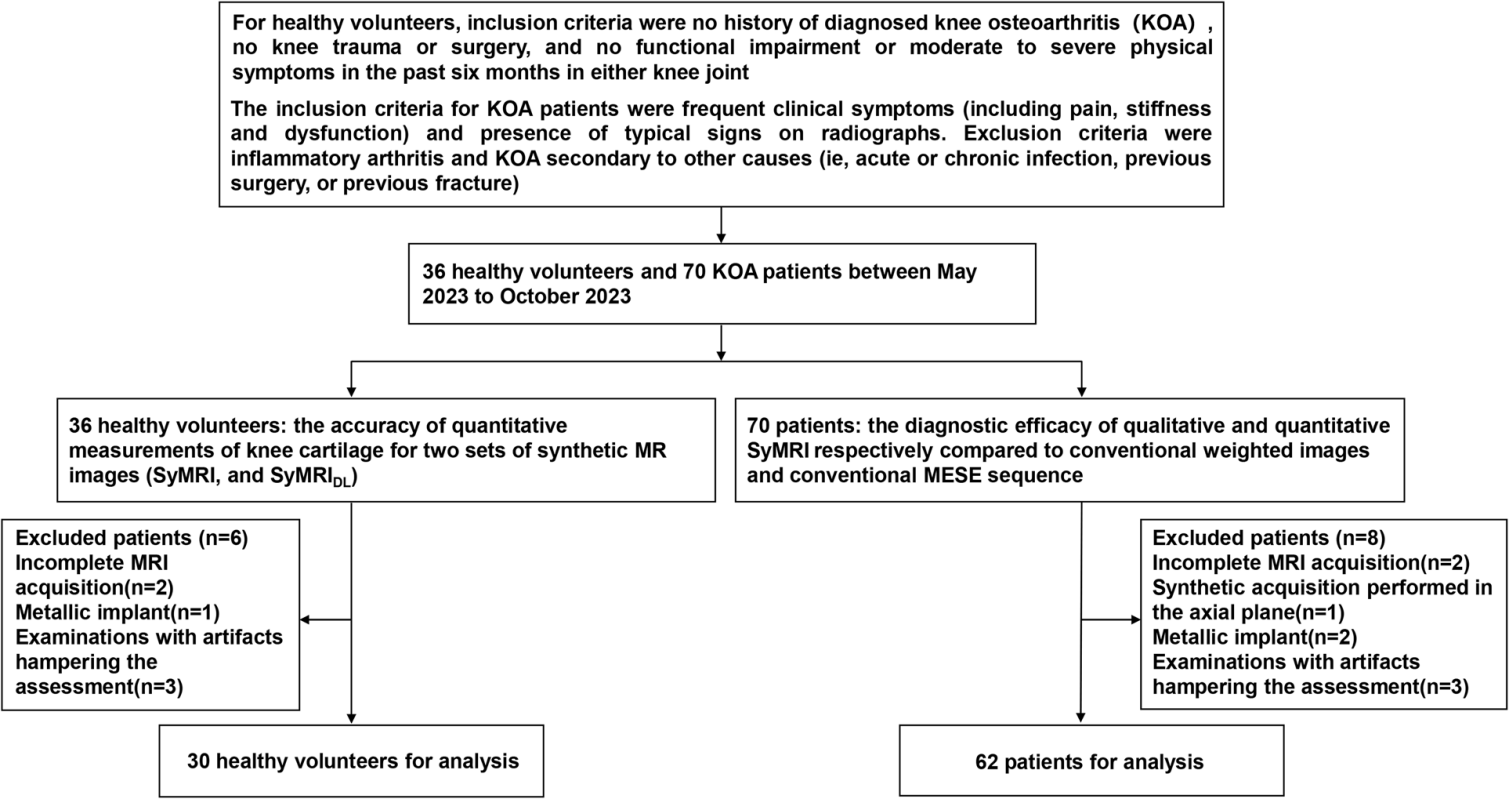
The flowchart for subject inclusion and exclusion

In vivo experiments in this study contained two parts: (1) The accuracy of quantitative measurements of knee cartilage for two sets of synthetic MR images (SyMRI and SyMRI_DL_) was assessed among 36 healthy volunteers. All volunteers were free from any history of knee trauma or surgery and had no pain or swelling. In order to minimize the inclusion of individuals with early cartilage injuries, only healthy volunteers aged between 18 and 30 years were included. (2) The diagnostic performance of SyMRI_DL_ was assessed for patients with KOA using conventional MRI as standard reference. The inclusion criteria for KOA patients were frequent clinical symptoms (including pain, stiffness, and dysfunction) and presence of typical signs on radiographs. Exclusion criteria were inflammatory arthritis and KOA secondary to other causes (i.e., acute or chronic infection, previous surgery, or previous fracture) [14]. All subjects underwent standard standing anteroposterior knee radiographs and completed a standardized clinical WOMAC questionnaire for pain degree, functional impairment, and stiffness on a five-point scale (no, slight, moderate, severe, or extreme) before undergoing MR imaging [15]. Figure 1 shows subject inclusion and exclusion.

### Image assessment

Two musculoskeletal radiologists (K.W., L.L.), with 5 and 10 years of experience respectively, independently assessed the MR images. Both readers were blinded to all patient information and reconstruction methods. To limit the potential for recall bias, interpretations of the conventional and synthetic weighted images (with and without DLR) were separated by at least 2 weeks for each participant. Additionally, the readers were blinded to each other’s assessments. The subjective assessment of knee on synthetic T1-, PD-, STIR- weighted images and conventional T1-, PD-, STIR-T2-weighted images were evaluated for overall image quality, artifacts, sharpness and subjective SNR using a 5-point Likert scale (1 = non-diagnostic; 2 = low image quality; 3 = moderate image quality; 4 = good image quality; 5 = excellent image quality).

The MRI OA Knee Score (MOAKS) [16] of 13 joint features, as listed in Table 2, was also evaluated according to previous guidelines [17]. Knee MOAKS scores were also performed on DLR synthetic MRI MOAKS, and the inter-reader agreement was analyzed using the Cohen Kappa. Both positive and negative percentage agreement (PPA, NPA) were calculated as the sensitivity and specificity compared to conventional MRI. Following the independent readings, a consensus was made and used to analyze reader discrepancies.

**Table 2:** The MOAKS assessment based on both conventional and DLR synthetic MRI and the accuracy of DLR synthetic MRI using the conventional MRI as standard reference

MOAKS feature	Positive finds	Total reads	Positive %	PPA	NPA	Cohen’s κ%
Cartilage thickness	66	868	7.6	92, 95	100, 100	86 (76–95)
Cartilage SA	99	868	11.4	96, 96	99, 99	83 (73–92)
Osteophytes	237	744	31.9	96, 100	99, 99	78 (71–85)
Meniscus tears	20	1116	1.8	90, 100	100, 100	0 (−99 to 99)
Meniscus signal	50	744	6.7	90, 96	98, 99	55 (10–99)
Meniscus extrusion	10	248	4.0	50, 70	98, 99	73 (35–99)
BML subregions	128	930	13.8	98, 100	97, 100	85 (78–93)
BML sizes	NA	NA	NA	94, 94	100, 100	81 (71–90)
Hoffa synovitis	23	124	18.5	65, 83	88, 82	49 (20–79)
Effusion synovitis	41	124	33.1	100, 100	100, 100	87 (72–99)
Ligaments	14	372	3.8	43, 64	100, 100	34 (0–76)
Other	12	186	6.5	83, 100	100, 100	0 (−99 to 99)
Total	828	7254	11.4	95	99	81 (78–85)

The total number of scored instances and the prevalence rate can be seen in the table. In addition, positive percentage agreement (PPA) and negative percentage agreement (NPA) for both readers (separated by commas), and the corresponding inter-reader Cohen’s κ percentage (with a 95% confidence interval) can also be seen. The Cohen’s Kappa value of 0% for meniscus tears is attributed to a small sample size of meniscus tears and dataset in non-normal distribution. The ‘Other’ category includes the features of the popliteal cysts, ganglion cysts, iliotibial band, and loose bodies

*Cartilage SA* cartilage surface area, *BML* bone marrow lesion

### Image processing and analysis

The conventional MESE and MAGnetic resonance image Compilation (MAGiC; GE Healthcare) sequences were analyzed respectively using the CartiGram on advanced workstation 4.7 (AW 4.7, GE Healthcare) and SyMRI (Linköping, Sweden, version 8.0.4) to generate T2 maps.

Two readers (X.L., H.L.) with 3 years of experience in musculoskeletal diagnosis independently measured the knee cartilage T2-relaxation time of the healthy volunteers included. To examine inter-reader agreement, T2 values were measured on media femoral condyle (MFC) and lateral femoral condyle (LFC), lateral tibial plateau (LTP), medial tibial plateau (MTP), patella (PAT), and trochlea (TRO) areas of the knee cartilage in a total of eight subregions. Using the posterior boundary of the meniscus as a landmark, central weightbearing (CMFC/CLFC) and posterior weightbearing (PMFC/PLFC) of MFC and LFC were further divided [18]. Eight regions of interest (ROIs) were manually delineated by two readers on two selected sagittal slices that cover the central areas of the lateral and medial condyles. Each ROI was carefully sketched to ensure that at least 2 voxels of thickness were included in the vertical direction of the cartilage to exclude voxels in adjacent tissues. If there was a slight movement of the knee between scans, the images were aligned using rigid transformations to ensure that each sequence represented the same physical location. However, significant movement in some cases were excluded for measure. T2 measurements of each ROI were averaged for each sequence.

To examine the diagnostic performance, the mean T2 values for four subregions (LFC, MFC, LTP and MTP) were calculated and compared between each two groups, where TRO and PAT areas were excluded accordingly for possibly magic angle effect and not belonging to the tibiofemoral cartilage. In this part, all subjects were classified into three groups according to the Whole-Organ MRI Score (WORMS) [19] and MOAKS scoring systems: (1) group 1: Cartilage grade 0 (normal thickness and signal intensity; MOAKS = 0 and WORMS = 0); (2) group 2: Cartilage grade 1 (normal thickness, but hyperintensity on T2-weighted images; MOAKS = 0 and WORMS = 1); (3) group 3: Cartilage grades > 1 (partial or full-thickness cartilage loss; MOAKS > 0 and WORMS > 1).

### Statistical analysis

SPSS (version 25.0, Chicago, IL) and Prism (9, GraphPad Software) were utilized for statistical analysis. Conventional MRI and T2_MESE_ served as the reference standards. Continuous variables were reported as mean ± standard deviation, while discrete variables were reported as median with interquartile range. For comparison between SyMRI, SyMRI_DL_, and the reference standards, one-way analysis of variance (ANOVA) with post hoc Tukey test was used for continuous variables, while the Friedman test with post hoc Bonferroni correction was applied to discrete variables. The intraclass correlation coefficient (ICC) with two-way random effects model was used to assess inter-reader agreement of T2-SyMRI, T2-SyMRI_DL_, and T2_MESE_ [20], whereas linear regression and Bland–Altman analysis were used to evaluate the correlation and agreement between T2 values derived from each image dataset. Cohen’s Kappa percentage (κ%) with the corresponding 95% confidence intervals (CI) showed inter-reader agreement of each MOAKS feature and image quality scores. Both positive and negative percentage agreement (PPA, NPA) were calculated as the sensitivity and specificity using conventional MRI as standard reference. Between-group differences in T2 values were assessed using ANOVA with post hoc Tukey test. A *p*-value < 0.05 indicated statistical significance.

## Results

### T2 estimation

**Fig. 2:**
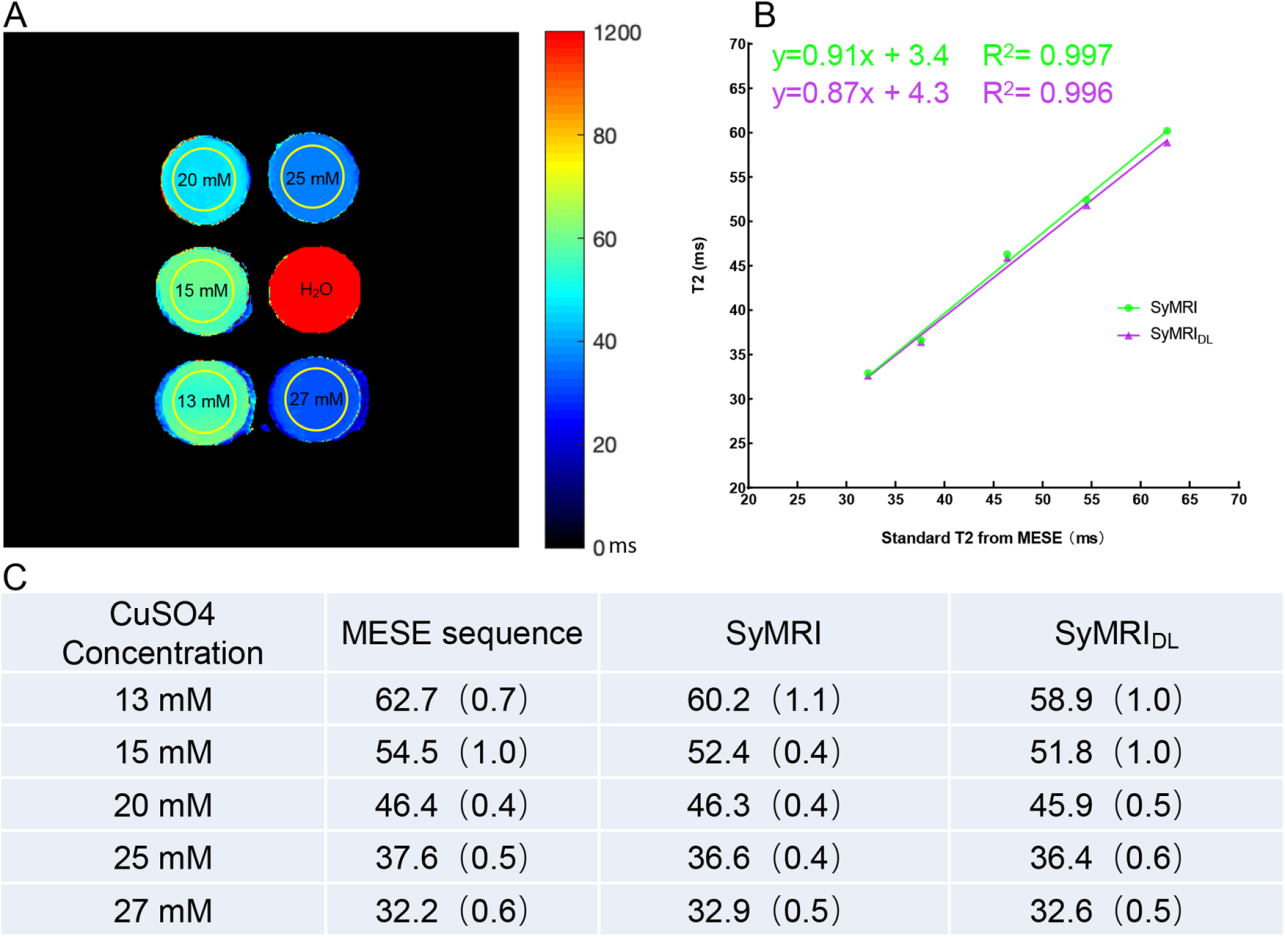
Estimated T2 values of each tube filled with specific concentrations of aqueous CuSO_4_ were acquired were obtained with MAGiC and MESE sequence. **A** A circular region of interest (25 mm diameter, yellow circle) was carefully positioned within each tube (28 mm diameter) in guarantee of signal homogeneity. **B** Scatter plot showed the correlation of T2-SyMRI and SyMRI_DL_ versus T2_MESE_. **C** T2-SyMRI, T2-SyMRI_DL_ and T2_MESE_ values at different concentrations were shown. All data were presented in mean (standard deviation)

Five tubes with copper sulfate solution at different concentrations showed no significant difference between T2-SyMRI and T2-SyMRI_DL_ measurements respectively (all *p* > 0.05). T2-syMRI and T2-syMRI_DL_ measurements were not significantly different from T2_MESE_ as standard T2 values (both *p* > 0.05) and had strong linear and robust relations (*R*^2^ > 0.99 for all, see Fig. 2).

Moderate to excellent inter-reader agreement (ICC, 0.65–0.92) was observed for both T2-SyMRI and T2-SyMRI_DL_ measurements (Supplementary Table 1). Bland–Altman analysis demonstrated minimal bias between readers for T2-SyMRI measurements (0.25 ms; 95% limits of agreement: −6.2, 6.8 ms) and T2-SyMRI_DL_ measurements (0.05 ms; 95% limits of agreement: −4.5, 4.6 ms).

**Fig. 3:**
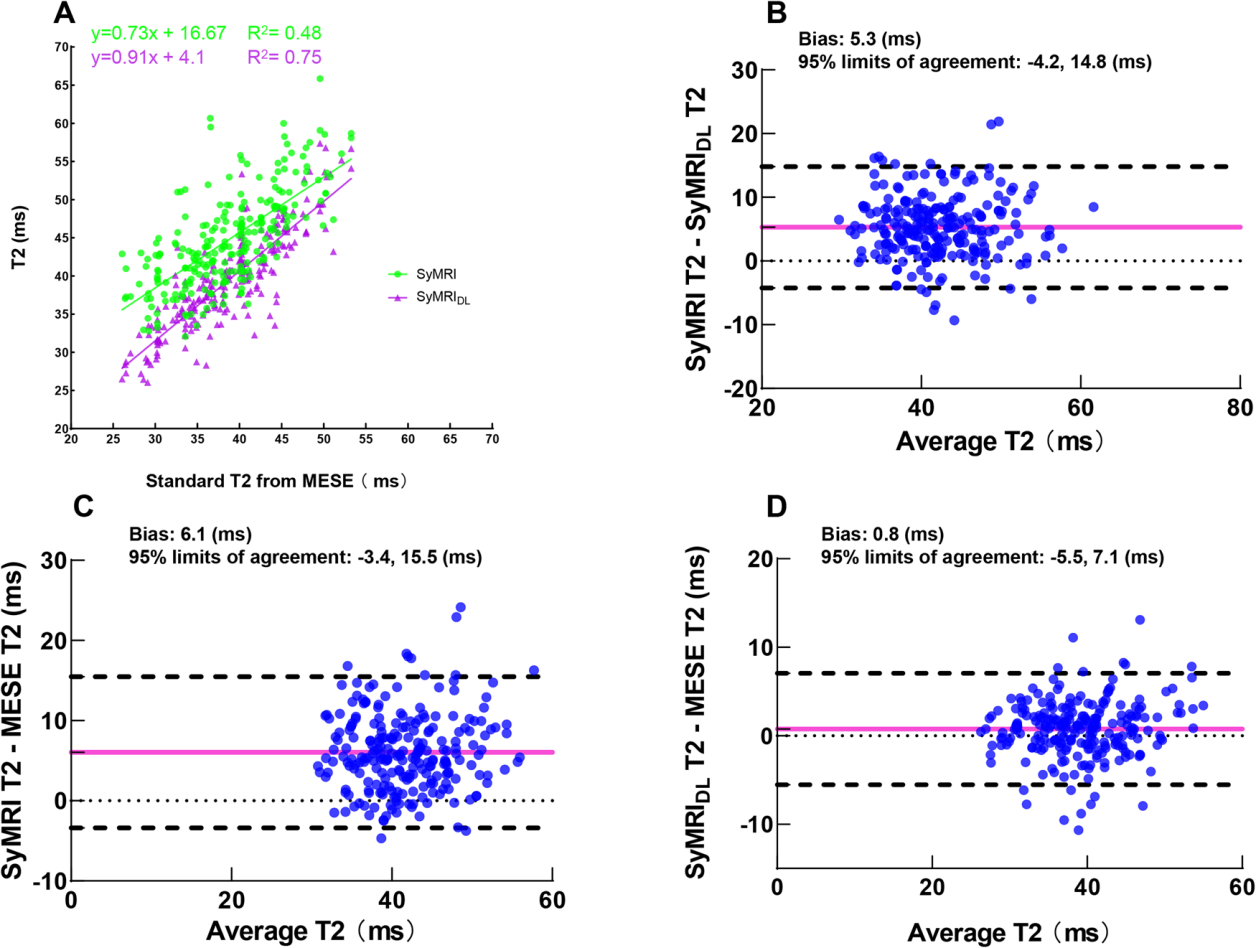
**A** Scatter plot displays T2-SyMRI and T2-SyMRI_DL_ against T2_MESE_ as standard reference. Regression analysis (continuous lines) indicates the intercept and slope of agreement. Bland–Altman plots for (**B**) T2-SyMRI versus T2-SyMRI_DL_, (**C**) T2-SyMRI versus T2_MESE_, (**D**) T2-SyMRI_DL_ versus T2_MESE_ with the mean bias (solid pink line) and the 95% limits of agreement (dotted black line)

Analysis of cartilage subregions in 30 healthy volunteers (11 males, aged 24 ± 3 years; 19 females, aged 22 ± 3 years) revealed significant differences between T2-SyMRI and T2-SyMRI_DL_ measurements (*p* < 0.001, Supplementary Table 2), with the mean bias of 5.3 ms (95% LOA: −4.2, 14.8) (Fig. 3B). T2-SyMRI measurements were significantly higher than T2_MESE_ measurements (*p* < 0.001), with the mean bias of 6.1 ms (95% LOA: −3.4, 15.5) (Fig. 3C). Conversely, no significant difference was observed between T2-SyMRI_DL_ and T2_MESE_ measurements (*p* > 0.05), with the mean bias of 0.8 ms (95% LOA: −5.5, 7.1) (Fig. 3D). Regression analysis demonstrated a stronger correlation between T2-SyMRI_DL_ and T2_MESE_ measurements (*R*² = 0.75) than between T2-SyMRI and T2_MESE_ measurements (*R*² = 0.48, Fig. 3A). Furthermore, T2-syMRI_DL_ (0.91) shower higher slope with T2_MESE_ than T2-syMRI (0.73).

### Qualitative assessment of DLR synthetic and conventional MRI

**Fig. 4:**
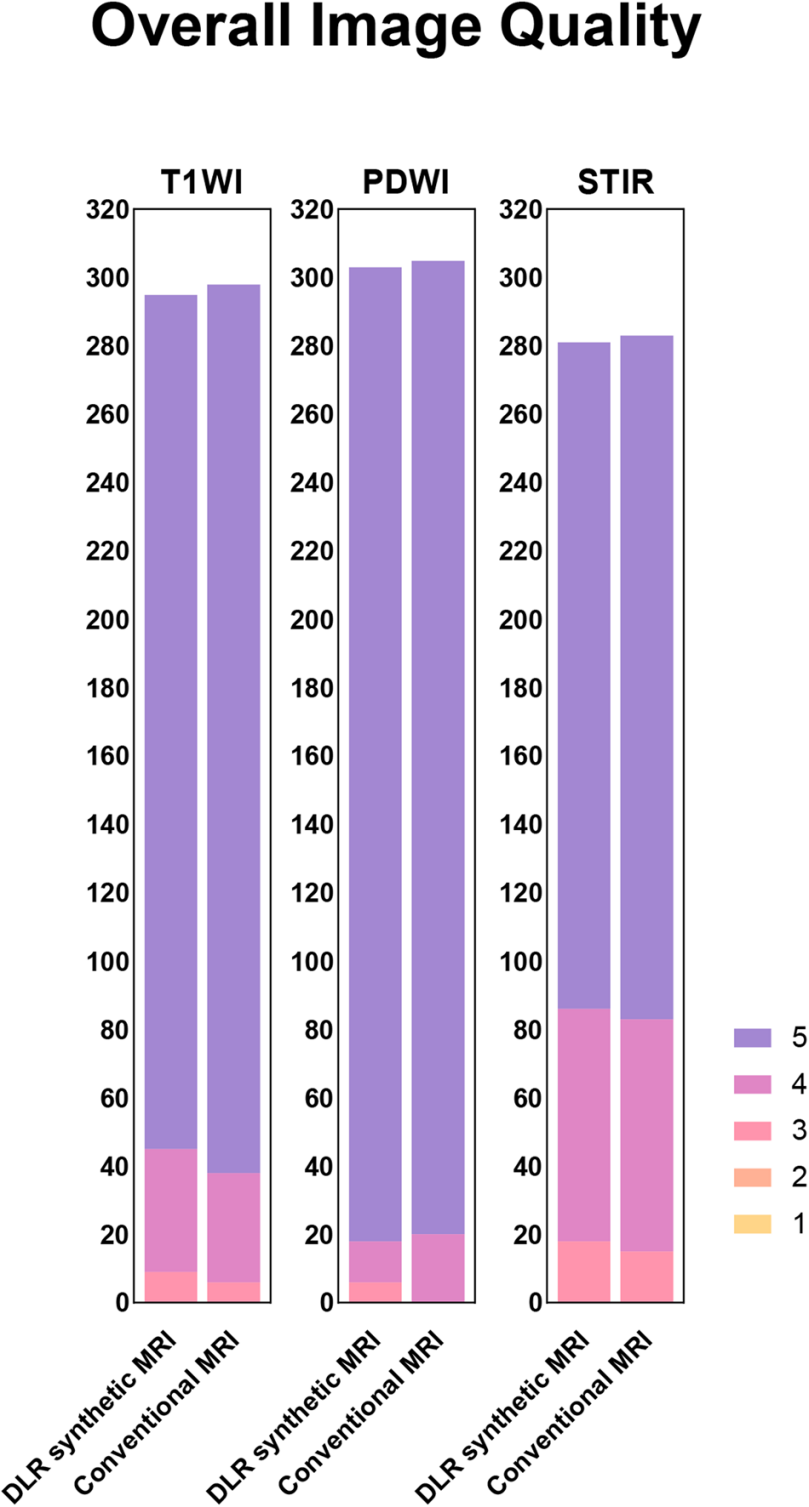
Comparisons of the overall image quality among different weighted images acquired with conventional and DLR synthetic MRI

62 patients (31 females, 53.1 ± 11.7 years; 31 males 52.5 ± 11.9 years) met the inclusion criteria and were included for final analysis. In terms of qualitative assessment on synthetic contrast images using SyMRI_PI=2-DL_, all were classified as 18 controls (KL = 0, mean age 38.3 ± 6.9 years), 26 patients with mild OA (KL score = 1 or 2, mean age 59.0 ± 7.5 years), and 18 patients with severe OA patients (KL score = 3 or 4, mean age = 58.2 ± 7.1years) according to the Kellgren-Lawrence (KL) grade and OA severity assessed by the standard standing anteroposterior radiographs of knee [21]. With substantial to almost perfect inter-reader agreement (kappa, 0.62–0.88), SyMRI_DL_ showed significantly higher ratings of subjective evaluation including overall image quality, sharpness, and subjective signal-to-noise ratio (SNR) than SyMRI for all contrast weightings (*p* < 0.001); however, the perceived extent of artifacts did not differ (*p* > 0.05). In comparison of conventional MR images to SyMRI_DL_, no significant differences in overall image quality, sharpness, artifacts, and subjective SNR were observed for all contrast weightings (all *p* > 0.05, Fig. 4, Supplementary Table 3).

**Fig. 5:**
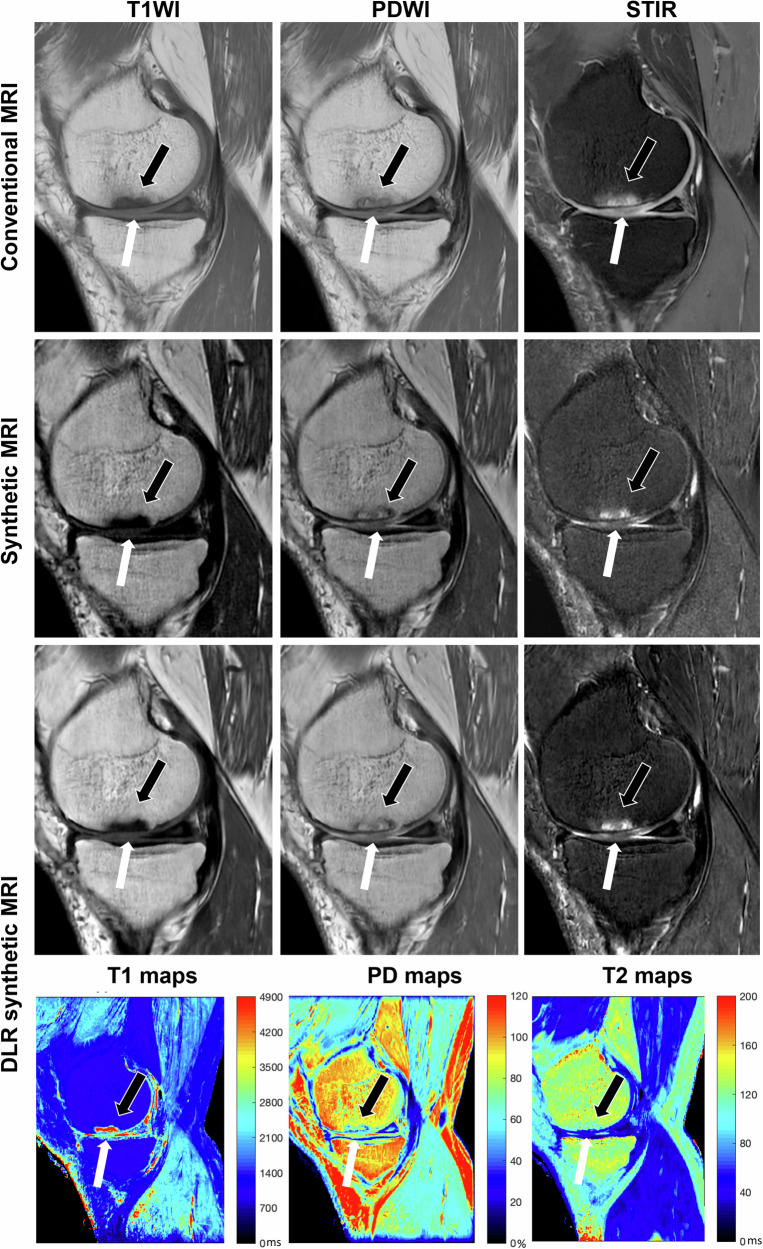
A representative image set of a 68-year-old man with right knee pain. Sagittal conventional and DLR synthetic knee MR images showed an inhomogeneous lesion both hypointensity and hyperintensity (white arrow) in the media femoral condyle accompanied by bone marrow edema (black arrow) while non-DLR SyMRI appeared vague lesion margins and more noises. STIR, short-tau inversion recovery; PD, proton density

In terms of diagnostic performance, inter-reader agreement on 13 MOAKS features was 81% (78–85% CI). Table 2 showed 95% (93–96% CI) and 99% (99–99% CI), respectively, for PPAs and NPAs of MOAKS. Cartilage loss, osteophytes, meniscal degeneration, and tears were equivalently observed between the two datasets (Fig. 5). The PPA was lowest in meniscus extrusion (60%) and ligaments (54%) and 8 out of 23 cases (35%) for Hoffa’s synovitis.

### Diagnostic performance of quantitative T2 maps using SyMRI

The number of cases with lesions in each subregion for 62 patients was classified into groups 1, 2, and 3 according to WORMS and MOAKS scoring systems (Supplementary Table 4); the average T2 measurements of each subregion and the significant inter-modality differences were labeled (Table 3). For the differentiation of subjects with and without normal-appearing cartilage, the medial tibial plateau was excluded due to the absence of normal thickness but hyperintensity on T2-weighted images. Higher T2-SyMRI, T2-SyMRI_DL_ and the T2_MESE_ measurements across all subregions (LFC, MFC, LTP and MTP) in group 3 than group 0 and group 1 (both *p* < 0.05). The majority of cartilage compartments showed statistically different T2-SyMRI_DL_ and the T2_MESE_ measurements between group 0 and group 1 (*p* < 0.05), while only the LTP exhibited statistically different T2-SyMRI measurements between grade 0 and grade 1 (*p* = 0.04).

**Table 3:** Average cartilage T2 values for groups with different WORMS and MOAKS scores

	LFC	MFC	LTP	MTP
MESE T2 values (ms)				
Group 1	36.9 ± 2.0	38.2 ± 1.7	33.9 ± 3.4	38.4 ± 3.2
Group 2	39.4 ± 6.2	**42.2 ± 3.8**^a^	**40.7 ± 4.7**^a^	—
Group 3	**50.4 ± 4.2**^a,b^	**50.9 ± 4.6**^a,b^	**47.1 ± 3.8**^a,b^	**49.7 ± 5.1**^a^
SyMRI_DL_ T2 values (ms)				
Group 1	37.9 ± 3.3	37.6 ± 4.9	35.9 ± 4.1	39.9 ± 4.2
Group 2	40.9 ± 5.0	**41.6 ± 3.9**^a^	**40.9 ± 4.9**^a^	—
Group 3	**49.8 ± 4.5**^a,b^	**51.4 ± 4.8**^a,b^	**48.3 ± 4.0**^a,b^	**50.8 ± 5.0**^a^
SyMRI T2 values (ms)				
Group 1	41.7 ± 3.8	43.1 ± 6.7	40.0 ± 4.7	45.0 ± 6.0
Group 2	44.6 ± 7.4	46.7 ± 5.6	46.0 ± 7.6^a^	—
Group 3	**55.1 ± 7.3**^a,b^	**57.5 ± 6.3**^a,b^	**55.0 ± 7.1**^a,b^	**56.6 ± 7.7**^a^

Data are reported as mean ± standard deviation. Significant values are shown in bold

*LFC* lateral femoral condyle, *MFC* media femoral condyle, *LTP* lateral tibial plateau, *MTP* medial tibial plateau, *SyMRI* synthetic MRI, *SyMRI*_*DL*_ deep-learning reconstruction synthetic MRI, *MESE* multi-echo spin echo

^a^ Significantly different to cartilage T2 values of group 1 (*p* < 0.05)

^b^ Significantly different to cartilage T2 values of group 2 (*p* < 0.05)

## Discussion

The study showed that DLR synthetic MRI provides qualitative morphometry comparable to conventional MRI, especially in terms of high PPA and NPA values using MOAKS scoring. For synthetic T2 measurements acquired with acceleration factors 2, DLR markedly minimized differences between synthetic and standard T2 values, further helpful for identifying early changes of cartilage in patients with normal-appearing cartilage. In other words, DLR synthetic MRI could serve as a promising tool for diagnosis and follow-ups in clinical practice, particularly beneficial for patients with knee cartilage degeneration.

DLR significantly mitigated noise and truncation artifact across all synthetic weighted images and parametric maps, thereby breaking the trade-off among SNR, acquisition time, and spatial resolution (i.e., acquisition of clinically preferred spatial resolution with subjectively acceptable image quality), enhancing the sharpness of anatomical visibility and grading accuracy [10, 12]. To balance clinically acceptable acquisition time with higher SNR, knee SyMRI was acquired with a slice thickness of 5 mm. However, this resulted in partial volume effects, leading to the omission of 16% of mild cartilage lesions on synthetic T2-weighted images, ultimately reducing diagnosis confidence and limiting its clinical utility [9]. Despite moderate to perfect inter-reader agreement (78–85%) and the equivalent performance of SyMRI_DL_ compared to conventional MR images on MOAKS features except meniscus extrusion (60%) and ligaments (54%), the low accuracy for both was attributed to the lack of synthetic coronal contrast-weighted images and the low prevalence rate in our recruited patients. The PPA for Hoffa’s synovitis was lower as the radiologist failed to identify the presence of synovitis for 8 out of 23 cases (35%). Synthetic DIR imaging may serve as a viable alternative to no gadolinium-enhanced MRI and might improve detection rates [7]. Similar to a previous finding of an increase in ghosting artifacts in myocardial delayed gadolinium-enhanced imaging with DLR [22], pulsation artifacts in DLR knee SyMRI were comparable or became more pronounced.

The phantom T2 estimation showed no significant difference between any two parametric maps (T2-SyMRI, T2-SyMRI_DL_, and T2_MESE_). Moreover, synthetic T2 values were closer to the standard T2 values when the CuSO_4_ concentrations were higher [23]. On the other hand, in vivo non-DLR synthetic T2 values were significantly higher than conventional ones [9, 24], attributing to fewer number of TE used in synthetic MRI (two versus eight) and the properties (e.g., conductivity and permittivity) of in vivo hyaline cartilage [9, 25]. Heteroscedastic variation occurs for resistance of coils and scanned objects as Gaussian noise exists especially at longer repetition and echo times [8, 26]. A model using logarithmic transformation and quadratic and segmented quadratic equations is utilized to correct heteroscedastic variation and residual errors of the quantitative data but remains still in synthetic MRI [27, 28]. We found DLR significantly narrowed the differences between SyMRI and standard T2 values, and there was no significant difference in T2 values between SyMRI_DL_ and T2_MESE_ measurements. Deep learning reconstruction alleviates the impacts of parallel imaging (acceleration factor of > 1) on signal loss, improves the visibility of anatomic structures on different contrast-weighted images due to the removal of unnecessary k-space information (e.g., noise), enhanced image sharpness, and obtains more repeatable quantitative values [29]. In addition to SNR, spatial resolution also plays a critical role in the accuracy of T2 measurements [30]. Higher inter-reader agreement for T2-SyMRI_DL_ across all cartilage regions compared to T2-SyMRI, likely for SyMRI_DL_ with higher spatial resolution and thinner slice thickness elevates between-tissue margins and minimizes partial volume effects for ROI sketch. This leads to more reliable T2 estimations.

Both conventional MESE and T2-SyMRI (with and without DLR) demonstrated a trend of increasing T2 values in the presence of cartilage defects [1, 31]. For detecting early cartilage degeneration, T2-SyMRI_DL_ and T2_MESE_ exhibited a broader T2 value dynamic range than T2-SyMRI, enabling the detection of subtle alterations in cartilage compositions over time as reported [1]. In our study, there were no statistically significant differences of T2-SyMRI_DL_ and T2_MESE_ for the lateral femoral condyle between normal-appearing and damage-visible cartilages, implying evaluation of articular cartilage should not rely on T2 mapping alone in either clinical practice or osteoarthritis research. Using synthetic MRI (synthetic weighted images and quantitative maps), this might explain controversial issues regarding an increase in T2-relaxation time in cartilage degeneration which results from higher water content and disruption of the collagen matrix, unchanged or even decreased T2-relaxation time in vitro degrading cartilages where depends on the amount of collagenase-induced collagen cleavage [32], and reduced internal signal intensity on T2-weighted fast spin-echo images for surgically confirmed cartilage lesions [33, 34].

Overall, the speed, accuracy, and precision of this approach are encouraging toward gaining a deeper understanding of clinical knee assessments. With the notable reduction in scan time of approximately 54%, SyMRI_DL_ could serve as an alternative to conventional high-resolution sagittal knee images for displaying morphology (characterization of articular abnormalities) and can also provide quantitative maps. In contrast to the observation of osteophytes and joint space narrowing at advanced disease stages using radiographs, SyMRI_DL_ has potential in early detection and long-term monitoring of cartilage changes, with the benefit of increased patient comfort and reduced motion artifact among up to 30% of inpatient and emergency and 7.5% of outpatient exams who undergo conventional imaging sequences [35].

Our study has several limitations. First, the study population is relatively small and only acquired on a 3.0-T MRI scanner from a single institution, confining our results to a specific magnetic field strength and limiting generalization, as well as suggesting a need for further research across various MRI settings. Second, arthroscopy was not employed as a validation metric due to its inability to assess all MOAKS features. The evaluation of MOAKS features using conventional MRI may result in errors, necessitating a larger cohort with KOA for validation. Third, there is a need to divide the knee cartilage into the deep and superficial layers because the signal intensity of both vary distinctly [30]. Lastly, reader confidence for determining the presence or absence of cartilage lesions between the SyMRI and SyMRI_DL_ was not assessed due to the relatively low image quality of SyMRI and high bias for repeated diagnosis of each reader.

In conclusion, we validated the feasibility of DLR SyMRI with an acceleration factor of 2 in the early detection of cartilage injury for patients with KOA. Future work should focus on the development of three-dimensional acquisition techniques to allow for high-resolution isotropic quantitative maps and morphologic images.

## Supplementary information

Supplementary Tables

## Data Availability

The data generated during the current study are available from the corresponding author upon reasonable request.

## Abbreviations

DL: Deep learning
IW: Intermediate weighted
MESE: Multi-echo spin echo
PD: Proton density
SNR: Signal-to-noise ratio
SyMRI: Synthetic MR imaging
SyMRIDL: DL-reconstructed synthetic MRI
