# Supplementary material for: Super-resolution synthetic MRI using deep learning reconstruction for accurate diagnosis of knee osteoarthritis
Source: Insights Imaging. 2025 Feb 17;16:44. doi: 10.1186/s13244-025-01911-z (PMC11832993; doi:10.1186/s13244-025-01911-z)
Supplement: Supplementary file 1 — Supplementary Tables [file 13244_2025_1911_MOESM1_ESM.pdf]

Super-Resolution Synthetic MRI using Deep Learning Reconstruction for Accurate Diagnosis of Knee Osteoarthritis

ELECTRONIC SUPPLEMENTARY MATERIAL

Supplementary Table 1: The inter-reader agreement on two of parametric maps (SyMRI with/without DLR and the conventional MESE)

Region	MESE sequence	SyMRI	$SyMRI_{DL}$
PAT	0.77 (0.57-0.88)	0.68 (0.43-0.83)	0.90 (0.80-0.95)
TRO	0.89 (0.79-0.95)	0.71 (0.47-0.85)	0.84 (0.68-0.92)
LTP	0.95 (0.89-0.97)	0.65 (0.38-0.82)	0.80 (0.63-0.90)
CLFC	0.88 (0.77-0.94)	0.74 (0.53-0.89)	0.71 (0.48-0.85)
PLFC	0.94 (0.89-0.97)	0.88 (0.77-0.94)	0.86 (0.72-0.93)
MTP	0.94 (0.88-0.97)	0.73 (0.51-0.86)	0.92 (0.84-0.96)
CMFC	0.81 (0.65-0.91)	0.83 (0.66-0.91)	0.88 (0.77-0.94)
PMFC	0.72 (0.48-0.86)	0.67 (0.41-0.83)	0.72 (0.48-0.85)

Note—Data are ICC value and 95% confidence intervals in parentheses. ICC = intraclass correlation coefficient. SyMRI = synthetic MRI, $SyMRI_{DL}$ = Deep-learning reconstruction synthetic MRI, MESE = multi-echo spin echo.

Supplementary Table 2: T2 values in different cartilage subregions on DLR or non-DLR synthetic MRIs and the conventional MESE

Region	MESE sequence	SyMRI	$SyMRI_{DL}$	SyMRI vs $SyMRI_{DL}$	SyMRI vs MESE	$SyMRI_{DL}$ vs MESE
				P-value	*P*-value	*P*-value
PAT	31.22±2.65	37.37±3.09	31.45±3.10	**< 0.001**	**< 0.001**	0.95
TRO	46.58±4.18	53.25±5.07	48.95±4.92	**< 0.001**	**< 0.001**	0.13
LTP	33.25±4.61	43.26±4.74	33.25±4.61	**< 0.001**	**< 0.001**	0.92
CLFC	37.99±3.30	43.26±2.28	39.16±3.42	**< 0.001**	**< 0.001**	0.31
PLFC	36.7±3.18	39.94±3.61	37.88±3.56	0.06	**< 0.001**	0.39
MTP	43.65±3.73	50.33±4.14	43.21±3.61	**< 0.001**	**< 0.001**	0.89
CMFC	40.74±4.45	47.65±3.52	42.09±3.71	**< 0.001**	**< 0.001**	0.38
PMFC	39.23±2.58	42.72±3.67	38.96±3.07	**< 0.001**	**< 0.001**	0.94
All	38.67±5.98	44.72±6.27	39.43±6.32	**< 0.001**	**< 0.001**	0.38

Note—PAT = patella, TRO = trochlea, LTP = lateral tibial plateau, CLFC = central lateral femoral condyle, PLFC = posterior lateral femoral condyle, MTP = medial tibial plateau, CMFC = central medial femoral condyle, PMFC = posterior medial femoral condyle. Data are reported as mean ± standard deviation. *P* values were calculated using one-way analysis of variance with the post hoc Tukey test. Significant values are shown in bold.

Supplementary Table 3: Image quality including inter-modality and inter-reader agreement of conventional and synthetic MRI

Sequence	Item	Reader 1				Reader 2				Inter-reader Agreement		
		SyMRI	$SyMRI_{DL}$	Conv.	Friedman *P* value	SyMRI	$SyMRI_{DL}$	Conv.	Friedman *P* value	SyMRI	$SyMRI_{DL}$	Conv.
T1WI	overall image quality	4 (3-4) [bc]	5 (5-5) [a]	5 (5-5) [a]	**< 0.001**	4 (3-4) [bc]	5 (4-5) [a]	5 (4-5) [a]	**< 0.001**	0.73 (0.58-0.87)	0.79 (0.61-0.96)	0.76 (0.56-0.96)
	artifacts	5 (4-5)	5 (4-5)	5 (4-5)	**< 0.001**	5 (4-5)	5 (4-5)	5 (4-5)	0.287	0.65 (0.46-0.84)	0.66 (0.48-0.96)	0.65 (0.47-0.85)
	sharpness	3 (3-3) [bc]	5 (4-5) [a]	5 (5-5) [a]	**< 0.001**	3 (2-3) [bc]	5 (4-5) [a]	5 (4-5) [a]	**< 0.001**	0.67 (0.48-0.86)	0.68 (0.50-0.86)	0.68 (0.48-0.87)
	Subjective SNR	3 (3-4) [bc]	5 (4-5) [a]	5 (4-5) [a]	**< 0.001**	3 (3-4) [bc]	5 (4-5) [a]	5 (4-5) [a]	**< 0.001**	0.72 (0.56-0.88)	0.80 (0.65-0.94)	0.85 (0.72-0.98)
PDWI	overall image quality	4 (4-4) [bc]	5 (5-5) [a]	5 (5-5) [a]	**< 0.001**	4 (3-4) [bc]	5 (5-5) [a]	5 (5-5) [a]	**< 0.001**	0.71 (0.55-0.86)	0.64 (0.35-0.92)	0.63 (0.34-0.92)
	artifacts	5 (5-5)	5 (5-5)	5 (5-5)	0.061	5 (4-5)	5 (4-5)	5 (4-5)	0.311	0.68 (0.48-0.89)	0.62 (0.38-0.85)	0.71 (0.50-0.92)
	sharpness	3 (2-4) [bc]	5 (4-5) [a]	5 (5-5) [a]	**< 0.001**	3 (2-4) [bc]	5 (4-5) [a]	5 (4-5) [a]	**< 0.001**	0.65 (0.49-0.80)	0.72 (0.56-0.88)	0.77 (0.61-0.92)
	Subjective SNR	3 (3-3) [bc]	5 (4-5) [a]	5 (4-5) [a]	**< 0.001**	3 (2-3) [bc]	4 (4-5) [a]	4 (4-5) [a]	**< 0.001**	0.66 (0.49-0.83)	0.73 (0.58-0.88)	0.77 (0.62-0.92)
STIR	overall image quality	3 (2-3) [bc]	5 (4-5) [a]	5 (4-5) [a]	**< 0.001**	3 (2-3) [bc]	5 (4-5) [a]	5 (4-5) [a]	**< 0.001**	0.78 (0.65-0.91)	0.88 (0.77-0.99)	0.87 (0.77-0.99)
	artifacts	4 (3-4) [c]	4 (3-5)	4 (4-5) [a]	**< 0.001**	4 (3-4) [c]	4 (3-5)	5 (4-5) [a]	**< 0.001**	0.75 (0.63-0.87)	0.71 (0.56-0.86)	0.65 (0.48-0.82)
	sharpness	3 (2-3) [bc]	5 (4-5) [a]	5 (4-5) [a]	**< 0.001**	3 (2-3) [bc]	5 (4-5) [a]	5 (4-5) [a]	**< 0.001**	0.62 (0.48-0.77)	0.78 (0.62-0.93)	0.83 (0.70-0.97)
	Subjective SNR	3 (3-4) [bc]	5 (4-5) [a]	5 (4-5) [a]	**< 0.001**	3 (3-4) [bc]	4 (4-5) [a]	4 (4-5) [a]	**< 0.001**	0.78 (0.65-0.93)	0.65 (0.49-0.81)	0.69 (0.53-0.85)

Note—Data are reported as median (interquartile range); inter-reader agreements are expressed as κ values with 95% confidence intervals in parentheses. SNR = signal-to-noise ratio, SyMRI = synthetic MRI, $SyMRI_{DL}$ = Deep-learning reconstruction synthetic MRI, Conv. = conventional MRI. Significant values are shown in bold.

[a] Post hoc Bonferroni test *P* < 0.05 versus SyMRI.

[b] Post hoc Bonferroni test *P* < 0.05 versus $SyMRI_{DL}$.

[c] Post hoc Bonferroni test *P* < 0.05 versus Conv..

Supplementary Table 4: Number of tibiofemoral subregions with cartilage lesions for 62 patients

Region	MOAKS=0 & WORMS=0	MOAKS=0 & WORMS=1	MOAKS>0 & WORMS>1
LFC	40 (65)	8 (13)	14 (23)
MFC	28 (45)	16 (26)	18 (29)
LTP	37 (60)	11 (18)	14 (23)
MTP	52 (84)	0 (0)	10 (16)

Note—Data are the number of lesions in each tibiofemoral subregion, and data in parentheses are percentages.
